# Supplementary material for: IRF3 Activation in Mast Cells Promotes FcεRI-Mediated Allergic Inflammation
Source: Cells. 2023 May 28;12(11):1493. doi: 10.3390/cells12111493 (PMC10252328; doi:10.3390/cells12111493)
Supplement: Supplementary file 1 [file cells-12-01493-s001.zip › cells-2302721-supplementary.pdf]

Supplemental figure and legends

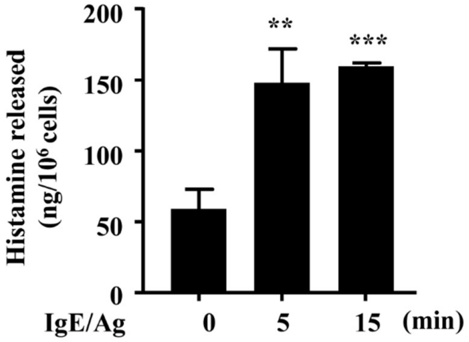

Figure S1. Level of histamine in DNP-HSA stimulated mBMMCs. Anti-DNP-IgE sensitized mBMMCs (5×10^5 cells/well in 12-well plates) were stimulated with DNP-HSA (100 ng/mL) for the indicated time. Histamine levels were detected with fluorometric histamine assay. The Graph represents the means ± standard deviation of the mean (n=3). $p^{**} < 0.01$, $p^{***} < 0.001$.

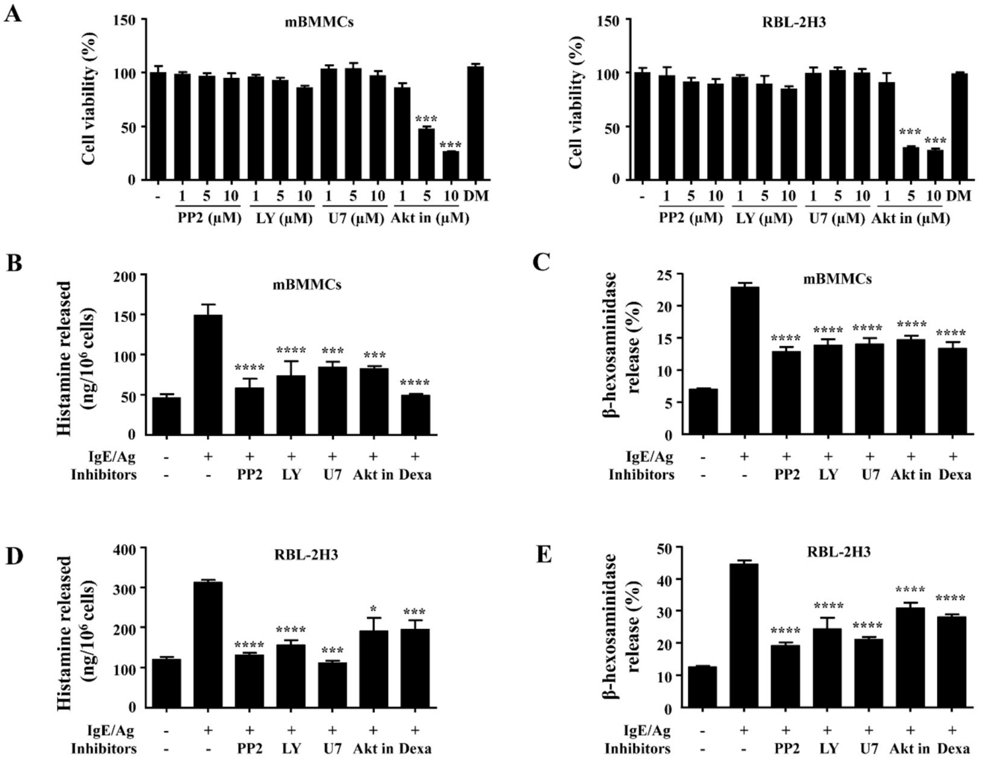

Figure S2. Effects of signaling inhibitors in mast cell degranulation. mBMMCs and RBL-2H3 (2×10^4 cells/well in 96-well plate) were treated with signaling inhibitors PP2, U 73122, LY 294002, and Akt inhibitor IV for 8 h and then incubated with MTT reagent. (A) The absorbance was detected using a spectrophotometer. For mast cell degranulation, anti-DNP sensitized mBMMCs and RBL-2H3 (5×10^5 cells/well in 12-well plates) were pretreated with or without PP2 (5 μM), LY 294002 (LY, 5 μM), U 73122 (U7, 5 μM), and Akt inhibitor IV (Akt in, 1 μM) or Dexa (10 μM) for 1 h and then challenged with DNP-HSA (100 ng/mL) for 30 min and 4 h, respectively. (B and D) Histamine levels were detected with fluorometric histamine assay in mBMMCs and RBL-2H3, respectively. (C and E) The level of β-hexosaminidase was measured using β-hexosaminidase substrate buffer in mBMMCs and RBL-2H3, respectively. Each data presented as a graph represents the means ± standard deviation of the mean (n=3). *Significant difference at $p^{*} < 0.05$, $p^{**} < 0.01$, $p^{***} < 0.001$ and $p^{****} < 0.0001$.. Dexa: dexamethasone.

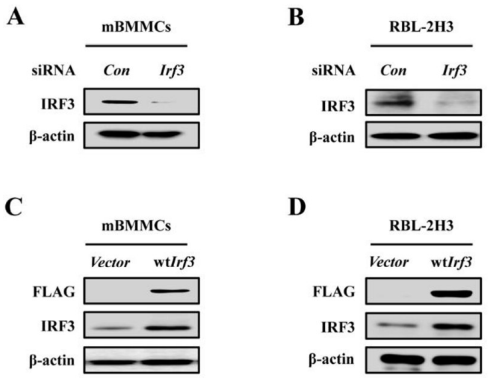

Figure S3. Knockdown and overexpression level of IRF3 in mBMMCs and RBL-2H3. mBMMCs (40 pmol/3 × 10^6 cells) and RBL-2H3 (40 pmol/2 × 10^5 cells) were transfected with control and *Irf3* siRNA for 24 h. The effectiveness of *Irf3* siRNA transfection was evaluated by Western blot analysis; (A) mBMMCs and (B) RBL-2H3. mBMMCs (0.8 μg/3 × 10^6 cells) and RBL-2H3 (1 μg/1 × 10^6 cells) were transfected with pCS107 vector and pCS107-IRF3 plasmids for 36 h and 24 h, respectively. Overexpression of IRF3 and FLAG-tagged protein expression were evaluated by Western blot analysis; (C) mBMMCs, and (D) RBL-2H3.

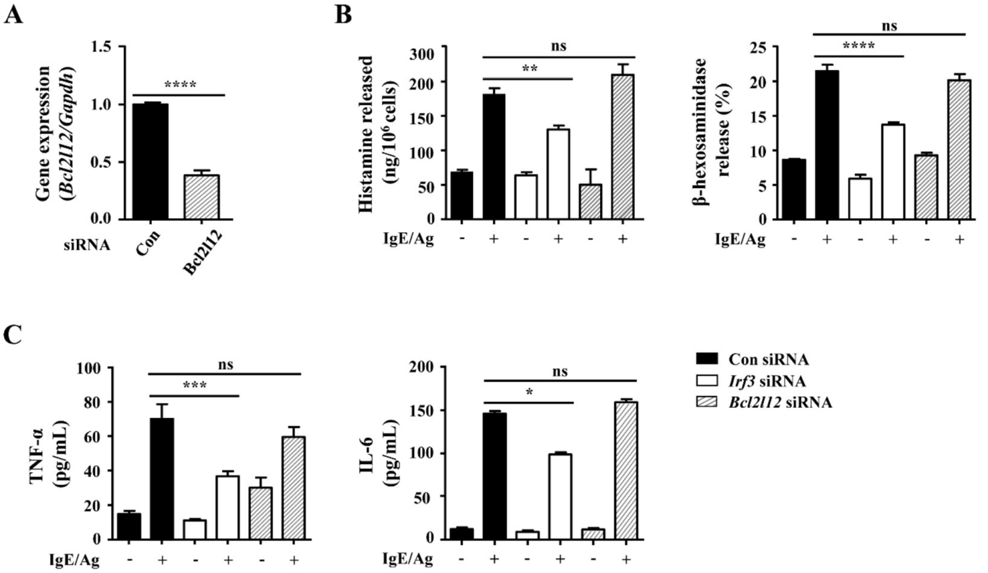

Figure S4. Effectiveness of *Irf3* and *Bcl2l12* knockdown in DNP-HSA stimulated mast cell mediators in mBMMCs. mBMMCs were transfected with control, *Irf3*, and *Bcl2l12* siRNA (40 pmol/3 × 10^6 cells) for 24 h. Then, transfected cells were sensitized with anti-DNP-IgE overnight and stimulated with DNP-HSA for 30 min for degranulation and for 8 h for ELISA. (A) Effectiveness of *Bcl2l12* siRNA by the time point. (B) Histamine levels were detected with a fluorescence plate reader. The level of β-hexosaminidase was measured using β-hexosaminidase substrate buffer. (C) The secretion of TNF-α and IL-6 was detected by ELISA. Each dataset presented as a graph represents the means ± standard error of the mean (n=3). *Significant difference at $p^* < 0.05$, $p^{**} < 0.01$, $p^{***} < 0.001$ and $p^{****} < 0.0001$..
